# Endoscopic ultrasound-guided fine needle core biopsy for the diagnosis of pancreatic malignant lesions: a systematic review and Meta-Analysis

**DOI:** 10.1038/srep22978

**Published:** 2016-03-10

**Authors:** Yongtao Yang, Lianyong Li, Changmin Qu, Shuwen Liang, Bolun Zeng, Zhiwen Luo

**Affiliations:** 1 Department of Gastroenterology, The 306^th^ Hospital of PLA, Chaoyang District, Beijing 100101, China

## Abstract

Endoscopic ultrasound-guided fine needle core biopsy (EUS-FNB) has been used as an effective method of diagnosing pancreatic malignant lesions. It has the advantage of providing well preserved tissue for histologic grading and subsequent molecular biological analysis. In order to estimate the diagnostic accuracy of EUS-FNB for pancreatic malignant lesions, studies assessing EUS-FNB to diagnose solid pancreatic masses were selected via Medline. Sixteen articles published between 2005 and 2015, covering 828 patients, met the inclusion criteria. The summary estimates for EUS-FNB differentiating malignant from benign solid pancreatic masses were: sensitivity 0.84 (95% confidence interval (CI), 0.82–0.87); specificity 0.98 (95% CI, 0.93–1.00); positive likelihood ratio 8.0 (95% CI 4.5–14.4); negative likelihood ratio 0.17 (95% CI 0.10–0.26); and DOR 64 (95% CI 30.4–134.8). The area under the sROC curve was 0.96. Subgroup analysis did not identify other factors that could substantially affect the diagnostic accuracy, such as the study design, location of study, number of centers, location of lesion, whether or not a cytopathologist was present, and so on. EUS-FNB is a reliable diagnostic tool for solid pancreatic masses and should be especially considered for pathology where histologic morphology is preferred for diagnosis.

Pancreatic cancer is a devastating disease with a poor prognosis, which is partially due to delayed diagnosis because of the late onset of symptoms^1^. Despite the many advancements that have been made in medical therapy in the past decade, there are still limited treatment modalities for advanced disease. Many epidemiologic surveys have shown that the 5-year survival rate is below 5%^2^,^3^. A significant proportion of patients could extend their survival time by surgery if their tumors were diagnosed at an early stage^4^. So early detection and accurate staging are crucial for the right treatment choice.

Tissue acquisition is of great importance to confirm diagnosis and guide treatment in pancreatic solid mass. The endoscopic ultrasound (EUS)-guided minimally invasive tissue acquisition techniques have become the standard of choice to sample pancreatic tissue that could only be biopsied through open techniques in the past^5^. The EUS method can detect lesions that are not seen by other imaging modalities and fine needle aspiration (FNA) is reported to be able to give a definitive cytological diagnosis^4^. A recent meta-analysis reported that the sensitivity and specificity of EUS-guided FNA (EUS-FNA) for pancreatic neoplasms were 85% and 98%, respectively^6^. The complication rate of EUS-FNA is approximately 1%–2%^7^. Having become widely accepted as safe and effective, EUS-FNA is considered a minimally invasive method of diagnosing pancreatic cancer^8^. Despite the widespread usage of EUS-FNA, one limitation related to this technique is that it often only provides a cytologic specimen with scant cellularity and lack of histologic architecture, which restrains us from making a complete tissue analysis for diagnosis and grade differentiation, especially for sarcomas or lymphomas^9^. As we know, in the era of molecular profiling and personalized oncologic therapies, a complete histologic sample for evaluation of molecular marker expression has become of paramount importance. Another limitation of EUS-FNA is the unclear number of passes required to achieve an adequate sample without an onsite cytopathologist^8^.

A fine needle biopsy (FNB) specimen containing core tissue may theoretically overcome the limitations associated with EUS-FNA and have a greater diagnostic accuracy, because it can provide well preserved cellular architecture for histological evaluation. To meet these expectations, many endeavors have been made to devise needles that could be compatible with an echoendoscope for obtaining a tissue core. Finally, different EUS-compatible core biopsy needles including a 19 gauge (G) Trucut biopsy needle, 19/22/25 G ProCore needle and nitinol-based flexible 19G needle have been developed and put into clinical practice based on different design mechanisms^10^,^11^,^12^,^13^. These needles make procurement of larger amounts of tissue available with preserved architecture for histological analysis. Many papers have reported the diagnostic accuracy of EUS-FNB for the differentiation of solid pancreatic masses, with a sensitivity and specificity ranging from 61–100% and 90–100%, respectively^14^. The objective of this meta-analysis was to evaluate the accuracy of EUS-FNB for the diagnosis of malignant solid pancreatic masses based on previous published literature.

## Results

**Figure 1:**
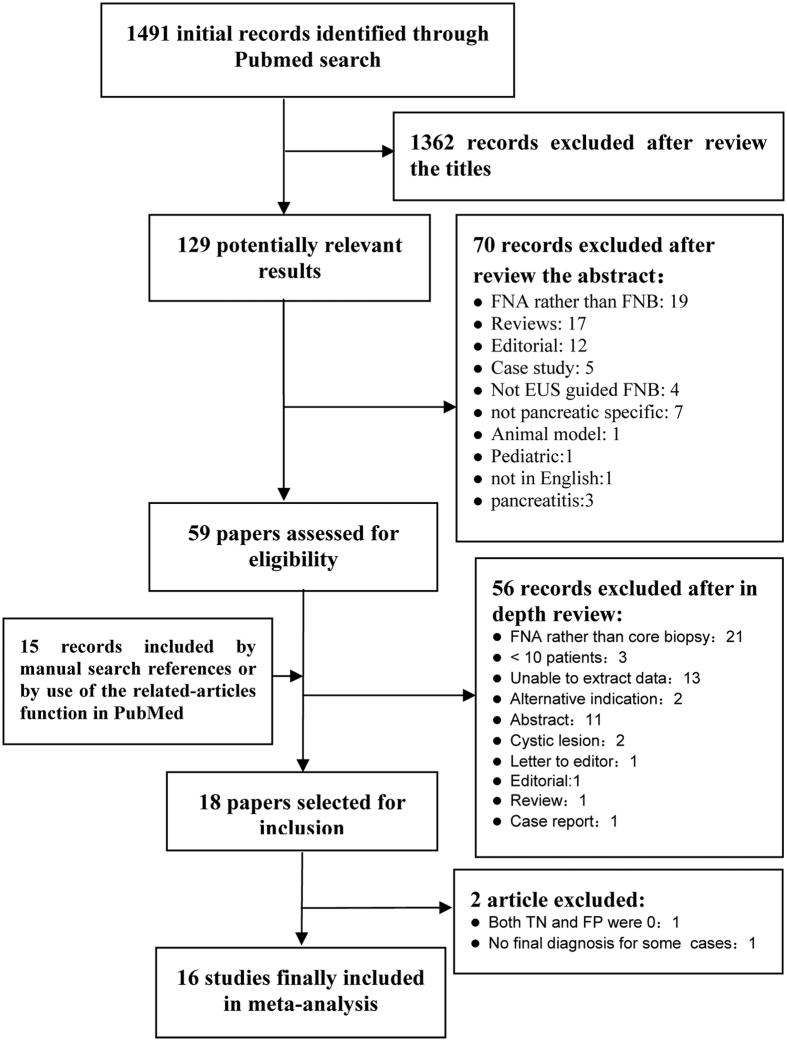
Flow chart of the study selection process for eligible studies in the systematic review. EUS, endoscopic ultrasound; FNA, fine needle aspiration; FNB, fine needle biopsy.

The Medline search, with preset search strategy, yielded 1491 records. Following an initial review of titles, 1362 records were excluded immediately. Seventy records were further excluded by abstract review for a variety of reasons, and the remaining 59 were left for full-text review. Manual review of the references of retrieved articles and the related-article function in PubMed identified 15 additional records concerning EUS-FNB in pancreatic mass lesions, of which most were excluded due to conference abstracts while three papers were included for full-text review 10,15,16. A further 56 records were excluded because they did not fit the criteria stated above, leaving 18 papers published between 2006 and 2015 that were selected in the meta-analysis. Finally, one paper was excluded as both the true negative and the false positive were zero^12^, and another one was excluded for the final diagnosis of some cases were not given^17^. Therefore, 16 studies were included in the final analysis^10^,^13^,^16^,^18^,^19^,^20^,^21^,^22^,^23^,^24^,^25^,^26^,^27^,^28^,^29^,^30^. Of the 16 papers, 12 were prospective studies, whereas the remaining four were retrospective studies. The median patient numbers included in the selected study was 50 (range 12–113). The detailed flow chart is shown in Fig. 1. The characteristics of included studies as well as QUADAS scores are described in Table 1.

All procedures of the studies were performed with standardized protocol by experienced investigators using either an Olympus (Olympus Medical Systems, Tokyo, Japan) or Pentax (Pentax Medical Company, Montvale, NJ, USA) convex or linear-array echoendoscope. The EUS-FNB procedure was performed with a 19-gauge, 22-gauge or 25-gauge Echotip ProCore biopsy needle (Wilson–Cook Medical, Winston-Salem, NC, USA), a flexible 19-gauge needle (Boston Scientific Corporation, Marlborough, MA, USA), or a 19 gauge QuickCore Trucut needles (Wilson–Cook Medical). Thirteen studies reported the inadequate or technial failures cases and atypical or suspicious cases separately. One study reported the inadequate or technial failures cases and atypical or suspicious cases in total. Two studies have such cases, but the exact numbers were unextractable (Table 2). Inadequate specimens were included in the assessment of sensitivity and specificity in the assessment of diagnostic performance. No serious complications were reported during any of the procedures. The pathological types of malignant pancreatic lesions included adenocarcinoma, solid-pseudopapillary tumor, malignant neuroendocrine tumor, acinar cell carcinoma, metastatic cancer, lymphoma and sarcoma. The benign solid pancreatic masses were mainly pancreatic inflammatory mass.

### Diagnostic accuracy

**Figure 2:**
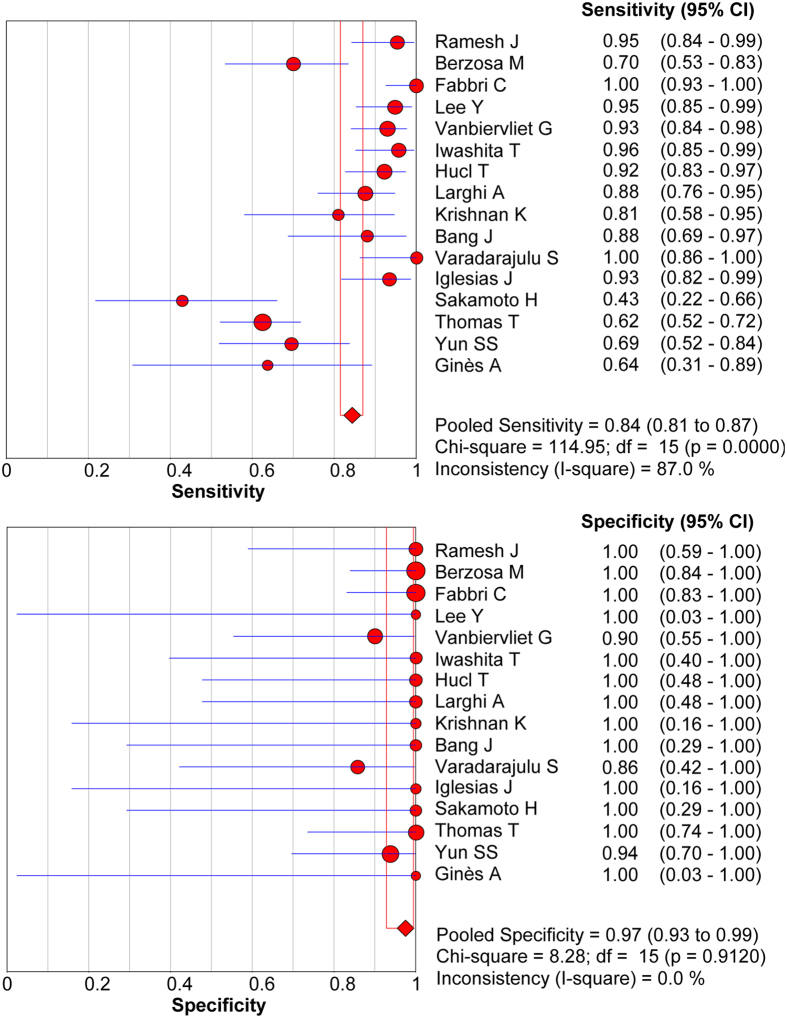
Forest plot of sensitivity and specificity estimates for EUS-FNB in the diagnosis of malignant pancreatic lesions. The point estimates of sensitivity and specificity from each study are shown as solid circles. Error bars indicate 95% CIs. EUS-FNB, endoscopic ultrasound-guided fine needle core biopsy; CI, confidence interval.

The forest plot of sensitivity and specificity of EUS-FNB for the diagnosis of malignant solid pancreatic mass is shown in Fig. 2. The sensitivity ranged from 0.43–1.00, whereas specificity was uniformly high and ranged from 0.90–1.00. The pooled sensitivity (random-effect model) and specificity (fixed-effect model) were 0.84 (95% CI, 0.82–0.87) and 0.98 (95% CI, 0.93–1.00), respectively. Significant heterogeneity was found in sensitivity (I^2^ = 87%), whereas it was not found in specificity (I^2^ = 0.0%). It was noted that the pooled PLR was 8.0 (95% CI 4.5–14.4), NLR was 0.17 (95% CI 0.10–0.26, Table 2) and DOR was 64 (95% CI 30.4–134.8, Table 2). The I^2^ values of PLR, NLR and DOR were 0.0%, 81.9% and 0.0%, respectively, indicating that there was no significant heterogeneity among studies regarding PLR and DOR, but not NLR.

**Figure 3:**
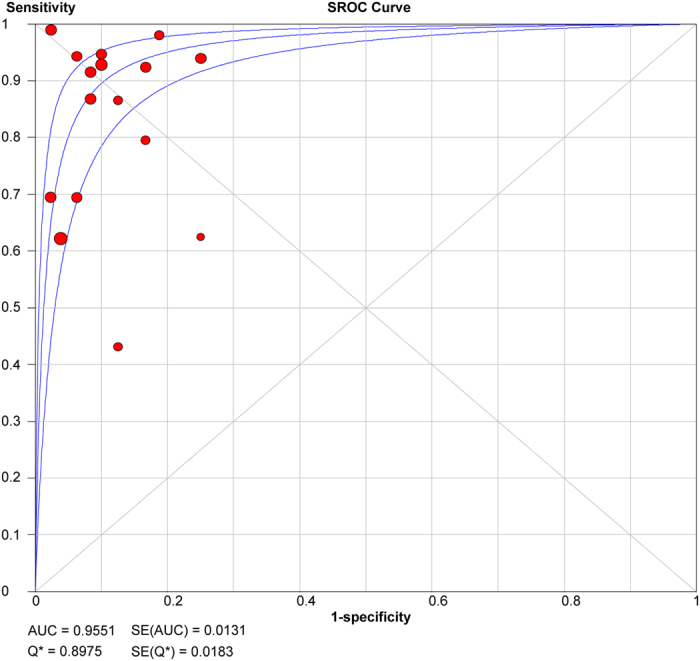
Summary receiver operating characteristic curves summarize the overall diagnostic accuracy of EUS-FNB in diagnosis of malignant solid pancreatic masses. EUS-FNB, endoscopic ultrasound-guided fine needle core biopsy.

The sROC curve of EUS-FNB diagnosing solid pancreatic lesions is shown in Fig. 3. The area under the sROC curve (AUC) is a method to assess the discriminating capability of a test. It represents an analytical summary of test performance. A higher AUC value means a better discriminating ability, a value of 1.0 means that the test has almost a perfect discrimination. The AUC value of EUS-FNB diagnosed malignant pancreatic lesions was 0.96 (SE = 0.013), indicating that the overall diagnostic accuracy of EUS-FNB was quite high. Index Q is another method to assess the diagnostic performance of an sROC curve. The index Q corresponds to the intersection point of the sROC curve with a diagonal line from the left upper corner to the right lower corner, in which sensitivity and specificity have a highest equal value. An index Q close to 1 means that the test has almost a perfect discrimination. The present Index Q value was 0.90, also suggesting a relatively high diagnostic accuracy.

### Subgroup analyses and meta-regression

Subgroup analysis based on factors potentially affecting the diagnostic accuracy is shown in Table 3. It was of note that needle type greatly influenced the diagnostic accuracy. The 19-gauge flexible needle had the highest sensitivity (97%) while the Trucut needle had the lowest sensitivity (62%). There was little difference among specificities. Although differences in diagnostic accuracy were observed and heterogeneity was reduced, the subgroups analysis could not adequately explain the heterogeneity with regard to sensitivity and NLR (all of the I^2^ were still above 50%).

The DOR is a single indicator of test accuracy that combines the data from sensitivity and specificity into a single entity^31^. The DOR equals the value of the PLR divided by the NLR. A higher value indicates a better discriminatory capability of a test^31^. To assess the effect of study characteristics, such as study design, single center versus multicenter, and so on, the DOR, meta-regression analysis was performed. Consistent with the subgroup analysis, although the DOR appeared improved in some subgroups, as shown in Table 3, none of the relative DOR (RDOR) reached statistical significance, demonstrating that the study characteristics, including study design, single center versus multicenter, and so on, did not significantly affect the diagnostic accuracy.

### Publication bias

**Figure 4:**
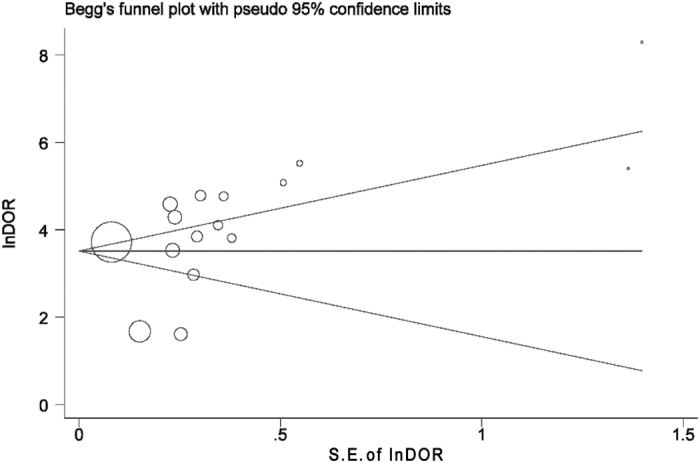
Funnel graph for the evaluation of potential publication bias of selected studies. Symbol size for each study is proportional to the study size. The line in the center indicates the summary DOR.The Egger test for publication bias was not significant.

Egger’s test and Begg’s funnel plot was used to analyze potential publication bias of the meta-analysis^32^. The Egger’s test showed a value of 2.20 (95% CI −2.04 ∼ 6.44, *P* = 0.285) on a per-patient analysis, and the Begg’s test showed a *P* value of 0.39 for the included studies (Fig. 4). These results indicated that there was no potential for publication bias.

## Discussion

The EUS-guided FNA has displaced surgical biopsy and became the standard practice of diagnosing pancreatic solid mass with the advantages of technical ease, lower cost, and decreased morbidity^33^. As the treatment modality is transiting to a more personalized approach, there is now an increased demand for additional tissue and histologic sections in addition to cytopathology from FNA specimens for the purpose of improved diagnostic accuracy and molecular characterization of tumors^8^. It is reasonable that larger-caliber or needles more specifically designed for obtaining core tissue would facilitate subsequent molecular biological analysis and histologic grading. However, unlike surgical resection specimens, EUS-FNB takes samples only from a very limited area of the suspected lesion, which enhances the chance of sampling and histologic interpretation errors due to tumor heterogeneity^9^. In fact, two studies have found sufficient tissue yielded high reproducibility and interobserver agreement while limited EUS-guided materials yielded significant discordance between the histologic and cytologic evaluations^34^,^35^. The actual benefit of EUS-FNB for pancreatic lesions still needs evaluation.

This meta-analysis has shown that EUS-FNB as a diagnostic tool for malignant pancreatic masses has a pooled sensitivity of 84% and a higher pooled specificity of 99%. We report an index Q value from the sROC of 0.9 and an AUC of 0.96, demonstrating a high degree of overall diagnostic accuracy. The DOR is used as an overall measure of the diagnostic accuracy of a diagnostic test. It is calculated as the odds of positivity among diseased persons, divided by the odds of positivity among non-diseased individuals^31^. The DOR value ranges from 0 to infinity, and a DOR value of 1.0 suggests that a test has no discriminability between patients with the disease and those without it^31^. In our study, we report a pooled DOR of 64.0, which also confirms a high level of overall diagnostic accuracy.

We also used both PLR and NLR as our measures of diagnostic accuracy for they can be more easily interpreted and applied to clinical practice^36^. A PLR value of 8.0 indicates that patients with malignant pancreatic mass have about a eight-fold higher chance of being EUS-FNB test-positive compared with those without malignant pancreatic mass. By comparison, the NLR was found to be 0.17, meaning that if the EUS-FNB test result was negative, the likelihood that this patient has malignant pancreatic mass is approximately 17%. So, on the basis of the currently available data, the PLR was high enough to be used as a valuable tool for rule-in diagnosis while the NLR was not sufficiently low enough to be used as a rule-out diagnosis.

As we have shown above, there was a significant heterogeneity with regard to sensitivity and NLR among the studies included. On subgroup analysis, based on several potential predefined sources of heterogeneity, we noted that differences in diagnostic accuracy occurred in different subgroups. However, heterogeneity was not adequately explained by analysis of these subgroups for all of them did not reach statistical significance (all I^2^ > 50%). Then, we used the meta-regression analysis to evaluate the effect of study characteristics, such as study design, study centers, etc., on RDOR. Consistently, with all of the factors, meta-regression was not statistically significant. We also analyzed potential publication bias according to the recommended guidelines. There was no significant publication bias found. We believed the variability in study population, research procedures and measurements may lead to heterogeneity in this study.

It had been reported that the complication rate for EUS-FNA was as low as 1% to 2%, with complications more usually occur when EUS-FNA was performed on cystic lesions than on solid lesions^37^. We found the EUS-FNB had a comparable complication rate with EUS-FNA. Of the studies included in this meta-analysis, the reported complications ranged from 0 to 7.5%. Only one study had greater than 5% complications. Examples of complications include self-limiting pancreatitis, infection, bleeding, abdominal pain requiring analgesics, aspiration pneumonia, and cholangitis due to biliary obstruction. No deaths or late complications related to EUS-FNB were reported.

Appropriate adjustment of the tip of the scope, avoiding puncture of vessels during operation, and use of antibiotics when pancreatic masses contain cystic components can help reduce FNB-related complications^38^. Theoretically, the size of the needle and the number of passes made may also influence the overall risk of complications. At present, no study has definitely evaluated whether the size of the needle can influence the risk of adverse events. Based on the very low overall rate of EUS-FNB complications, it is reasonable to recommend that a larger sample size needle should be used when necessary. However, it is also recommended that the diagnosis be made with the minimal number of passes to avoid unnecessary risks.

A few limitations of this meta-analysis should be mentioned. First, letters to the editors, conference abstracts, and non-English-language studies were excluded from this analysis and may have led to publication bias, although that was not statistically significant in this meta-analysis. However, we reviewed these letters and abstracts and found that the overall results were similar to the results in the included articles, which may reduce the potential of publication bias. Second, classifying the suspicious/atypical results on EUS-FNB as true positive may have led to overestimates of the diagnostic accuracy while classifying a few undiagnosed benign cases due to technique failure as false negative may have led us to underestimate the diagnostic accuracy. The rationale that we classified the suspicious/atypical as true positive was based on the conception that such a result could alert patient’s awareness of monitoring and subsequent imaging, which was of great usefulness for a better prognosis. Another important issue needed to address was that some studies had an unacceptably low sensitivity that couldn’t be easily explained by different populations. We believed that such low sensitivities mainly came from the high number of false negative due to the technical failure, especially the EUS-TNB needles were prone to fail to sample some lesions in the head of pancreas for the earlier studies. Also, the diagnostic accuracy was heavily reliant upon the experience of the endoscopists, studies performed at less experienced institutions tended to yielding lower sensitivities. However, the studies included in this analysis did not reflect the levels of experience at these institutions. Besides, malignant pancreatic mass is not always diagnosed by histologic analysis. It was diagnosed in some rare cases based just on the clinical course. With one study in our analysis the minimal clinical follow-up of just 5 months might lead to biased results.

In conclusion, EUS-FNB is a reliable and accurate diagnostic test for malignant pancreatic lesions, especially for the suspicion of pathology where histologic morphology was preferred for diagnosis. With improvements in technology, it could become the standard of choice. However, it should be interpreted in combination with clinical data and other conventional tests because the negative predictive value of this test is not high enough.

## Methods

### Search strategy and study selection

A comprehensive search of Medline (using PubMed as the search engine) was done to identify suitable studies up to May 2015. The search used a combination of terms (“biopsy” or “aspiration”) AND (“endoscopy” or “endoscopic”) AND (“pancreas” or “pancreatic”). The bibliographies of retrieved articles were searched to identify relevant studies manually. The related-articles function in PubMed was also used to further identify relevant articles. The search was not restricted to any particular language, but only articles written in English were retrieved for full evaluation. Data extraction and quality control were performed by two reviewers (YTY and LYL) for each selected study. Disagreements were resolved by making a consensus.

Studies included in the meta-analysis met the following criteria: (1) adult patients with suspected pancreatic solid mass; (2) final diagnosis was resolved by at least one of these criteria: (i) surgical diagnosis based on a resected specimen; (ii) typical histological or cytological characteristics of the EUS-FNA or EUS-FNB examination; (iii) clinical follow-up of at least five months for suspicion of benign pancreatic disease; (3) provided sufficient data to extract the diagnostic results such as true-positive, true-negative, false-positive, and false-negative; (4) solid pancreatic mass was the only lesion or contained other lesions but pancreatic mass cases were analyzed separately, and the number of patients with pancreatic lesions was over ten; and (5) written in English. The exclusion criteria were: (1) conference abstracts and letters to editors; (2) pediatric or animal studies; (3) assessing pancreatic cystic lesions; and (4) providing insufficient data to construct a 2 × 2 contingency table for calculating specificity and sensitivity.

### Data extraction and quality assessment

The cytological or histological results in some articles were reported as inadequate, benign, atypical, suspicious, or malignant. Then, we included atypical and suspicious cytology results as positive for malignancy, whereas we included cases of inadequate or technique failure as false negative when benign cases constituted only a very small fraction of total. Further information extracted from each article included: (1) publication year; (2) country of origin; (3) prospective or retrospective; (4) number of centers; (5) length of study; (6) number of benign and malignant patients; (7) needle types; (8) mean or median of passes; (9) lesion size; (10) lesion location; and (11) whether or not a cytopathologist was on site for all cases. To evaluate the study quality and potential for bias, an assessment was conducted using the Quality Assessment of Diagnostic Accuracy Studies (QUADAS) tool^39^. A total of 14 items were assessed for each study, with a maximum score 14. Disagreement between the two extracting authors was resolved by consensus.

### Statistical analyses

Standard methods recommended for meta-analyses of diagnostic test evaluations were used^36^. The estimates of diagnosis accuracy including sensitivity, specificity, positive likelihood ratio (PLR), negative likelihood ratio (NLR) and diagnostic odds ratio (DOR) were calculated for each study. Pooled results were constructed by using both the Mantel–Haenszel method (fixed-effect model) and the DerSimonian–Laird method (random-effect model) based on whether significant heterogeneity was absent or not^36^. The Cochrane Q test was used to estimate heterogeneity among the studies. Heterogeneity across the studies rather than from chance was expressed as inconsistency (I^2^), in the form of a percentage. An I^2^ above 50% was considered significant heterogeneity across the studies, which meant that the random-effect model rather than fixed-effect model method was used to calculate the pooled estimates^40^.

Summary receiver operating characteristic (sROC) analysis was performed based on the Moses and colleagues method^41^, which was used to reflect the discriminating ability of a diagnostic test. The sROC curve is a plot of the true positive rate (sensitivity) as a function of the false positive rate (1-specificity)^42^. An sROC curve is constructed based on a linear regression model to fit these points. The constructed linear regression equation to fit these points is as follows: D = β × S + α, where D = ln[TPR/(1−TPR)] − ln[FPR/(1−FPR)] and S = ln[TPR/(1−TPR)] + ln[FPR/(1−FPR)], and α is used as the y-intercept while β is the regression coefficient of independent variable S.

To explore the heterogeneity across the studies, subgroup analyses were performed according to: origin of study; study design (retrospective versus prospective); number of centers; location of lesion; and whether or not a cytopathologist was present for all cases. The variables in the subgroup analysis were used as covariates to perform meta-regression analysis to further explore potential sources of heterogeneity. We analyzed the effects of covariates on DOR according to the Moses–Shapiro–Littenberg model with recommended methods^43^. The publication bias for meta-analyses was analyzed using funnel plots and the Egger test, and was evaluated in the form of a funnel plot of standard error (SE) in the DOR (x) versus ln (DOR) (y).

The test accuracy, including sensitivity, specificity, PLR, NLR, DOR, sROC and meta-regression analyses, were performed using Meta-DiSc software (version 1.4)^44^. The publication bias analyses were performed using STATA software (version 12.0). Levels of significance were measured at *P* < 0.05.

## Additional Information

**How to cite this article**: Yang, Y. *et al.* Endoscopic ultrasound-guided fine needle core biopsy for the diagnosis of pancreatic malignant lesions: a systematic review and Meta-Analysis. *Sci. Rep.* **6**, 22978; doi: 10.1038/srep22978 (2016).

## Figures and Tables

**Table 1:** Characteristics of the selected studies.

Author	Year	Design	Country	Center	Time	Needle	ROSE	No.	Location	Masses (B/M)	Size(mm) Mean or median	Passes (mean or median)	QUADAS
Ramesh J	2015	P	USA	1	5	Flexible 19G	Yes	50	30/20	7/43	40.2	1.2	12
Berzosa M	2015	R	USA	2	13	22G ProCore	No	61	38/23	21/40	33	1.7	10
Fabbri C	2015	P	Italy	2	24	22G ProCore	No	68	32/36	20/48	16.5	1.5	11
Lee Y	2014	P	Korea	1	16	22G or 25G ProCore	Yes	58	24/34	3/55	36.5	1.3	13
Vanbiervliet G	2014	P	France	17	9	22G ProCore	No	80	50/30	10/70	33.9	NA	13
Iwashita T	2013	R	USA	1	7	25G ProCore	Yes	50	38/12	4/46	30.5	4	13
Hucl T	2013	P	India	1	16	22G ProCore	No	69	37/32	14/55	41.9	NA	12
Larghi A	2013	P	Multiple	5	2	22G ProCore	No	61	35/26	5/56	32.4	NA	12
Krishnan K	2013	R	USA	1	22	19G or 22G ProCore	Yes	23	NA	2/21	NA	NA	9
Bang J	2012	P	USA	1	3	22G PreCore	Yes	28	20/8	3/25	32.5	1.3	12
Varadarajulu S	2012	P	USA	1	3	flexible 19-G	Yes	32	32/0	7/25	34	1.4	11
Iglesias J	2011	P	Spain	5	4	19G PreCore	No	47	NA	45/2	NA	NA	10
Sakamoto H	2009	P	Japan	1	3	19G Trucut	Yes	24	12/12	6/18	32.8	NA	11
Thomas T	2009	P	UK	1	69	19G Trucut	No	113	NA	12/101	30	3	12
Yun SS	2007	R	USA	1	24	19G Trucut	No	52	30/22	12/40	NA	NA	9
Ginès A	2005	P	USA	1	6	19G Trucut	Yes	12	N/A	1/11	32.5	NA	11

P, prospective; R, retrospective; ROSE, rapid on-site cytopathology evaluation for all cases; No., Number; H/U, head or uncinate process of pancreas; B/M, benign/malignant; QUDAS, Quality Assessment of Diagnostic Accuracy Studies; NA, not available.

**Table 2:** Diagnostic performance (with 95% confidence intervals) derived from the 2 × 2 tables of individual studies.

Authors	Year	TP	FN	FP	TN	Inadequate/AS	Sensitivity	Specificity	PLR	NLR	DOR
Ramesh J	2015	41	2	0	7	4/0	95(84–99)	100(59–100)	15.1(1.0–221.2)	0.06(0.02–0.21)	249.0(10.8–5720.6)
Berzosa M	2015	28	12	0	21	16/0	70(54–83)	100(84–100)	30.6(2.0–477.3)	0.31(0.20–0.50)	98.0(5.5–1749.3)
Fabbri C	2015	48	0	0	20	10/4	100(93–100)	100(83–100)	41.6(2.7–643.0)	0.01(0.00–0.17)	3977.0(76.3–207333.1)
Lee Y	2014	54	3	0	1	0/0	95(85–99)	100(2.5–100)	3.8(0.3–41.5)	0.08(0.02–0.29)	46.7(1.6–1369.5)
Vanbiervliet G	2014	65	5	1	9	9/0	93(84–98)	90(56–100)	9.3(1.4–59.7)	0.08(0.03–0.19)	117.0(12.2–1118.3)
Iwashita T	2013	44	2	0	4	0/0	96(85–99)	100(40–100)	9.5(0.7–131.4)	0.06(0.02–0.20)	160.2(6.6–3880.9)
Hucl T	2013	59	5	0	5	5/0	92(83–97)	100(48–100)	11.0(0.8–156.2)	0.09(0.04–0.21)	119.0(5.8–2448.2)
Larghi A	2013	49	7	0	5	7/0	88(76–95)	100(48–100)	10.4(0.7–148.4)	0.14(0.07–0.29)	72.6(3.6–1451.2)
Krishnan K	2013	17	4	0	2	NA	81(58–95)	100(16–100)	4.8(0.4–60.5)	0.25(0.09–0.65)	19.4(0.8–481.0)
Bang J	2012	22	3	0	3	1/0	88(69–98)	100(29–100)	6.9(0.5–93)	0.15(0.05–0.43)	45.0(1.9–1071.3)
Varadarajulu S	2012	25	0	1	6	3	100(86–100)	86(42–100)	5.2(1.2 –22.1)	0.02(0.01–0.38)	221.0(8.0–6079.1)
Iglesias J	2011	42	3	0	2	2/0	93(82–99)	100(16–100)	5.5(0.4–69.7)	0.09(0.03–0.28)	60.7(2.4–1528.8)
Sakamoto H	2009	9	12	0	3	12/0	43(22–66)	100(29–100)	3.5(0.2–48.3)	0.65(0.39–1.09)	5.3(0.2–115.9)
Thomas T	2009	63	38	0	12	NA	62(52–72)	100(74–100)	16.2(1.1–246.3)	0.39(0.30–0.52)	41.2(2.4–716.4)
Yun SS	2007	25	11	1	15	0/5	69(52–84)	94(70–100)	11.1(1.6–75.0)	0.33(0.20–0.54)	34.1(4.0–291.2)
Ginès A	2005	7	4	0	1	2/0	64(31–89)	100(2.5–100)	2.5(0.2–28.7)	0.50(0.17–1.48)	5.0(0.2–150.9)
**Overall**		**598**	**111**	**3**	**116**		**84(82–87)**	**98(93–100)**	**8.0(4.5–14.4)**	**0.17(0.10–0.26)**	**64.0(30.4–134.8)**

FN, false negative; FP, false positive; TN, true negative; TP, true positive; Inadequate/AS, inadequate tissue or technical failure/ atypical or suspicious; PLR, positive likelihood ratio; NLR, negative likelihood ratio; DOR, diagnostic odds ratio; NA, not available.

**Table 3:** Subgroup analysis of diagnostic indices (with 95% confidence intervals) and subsequent meta-regression on DOR.

Subgroup	No. of studies	Sensitivity pooled	Specificity pooled	PLR pooled	NLR pooled	DOR pooled	RDOR	P Value
Study design								
Prospective	12	86(82–88)	97(91–100)	7.3(3.7–14.2)	0.14(0.07–0.27)	70(29–170)	1.29(0.18–9.12)	0.785
Retrospective	4	79(72–86)	98(88–99)	10.8(3.3–35.5)	0.24(0.14–0.41)	52(13–204)		
Study length								
<12	9	89(85–92)	95(84–99)	6.4(3.0–13.4)	0.14(0.06–0.30)	61(22–167)	2.08(0.34–12.75)	0.400
>12	7	80(76–84)	99(93–100)	11.5(4.5–29.3)	0.21(0.12–0.36)	68(23–205)		
Location								
USA	8	85(79–89)	97(89–100)	7.43(3.4–16.2)	0.19(0.12–0.33)	56(20–161)	115(0.22–5.98)	0.859
Others	8	84(81–87)	98(91–100)	8.85(3.7–21.1)	0.14(0.06–0.32)	73(25–214)		
Study center								
Single	11	81(77–85)	97(89–100)	6.65(3.3–13.1)	0.19(0.11–0.32)	45(18–110)	2.86(0.50–16.52)	0.218
Multiple	5	90(85–93)	98(91–100)	13.1(4.4–38.7)	0.12(0.05–0.27)	136(37–501)		
On–site pathol–ogist for all								
Yes	8	88(83–92)	96(82–100)	5.3(2.4–11.8)	0.16(0.06–0.38)	42(13–132)	3.77(0.64–22.33)	0.132
No	8	82(79–86)	98(92–100)	12.7(5.4–29.5)	0.17(0.09–0.29)	87(32–233)		
Needle type								
PreCore	10	91(88–93)	99(93–100)	9.3(4.3–20.6)	0.12(0.08–0.20)	95(36–247)	0.87(0.26–2.92)	0.811
Trucut	4	62(54–69)	97(84–100)	6.7(2.1–21.6)	0.43(0.32–0.57)	18(5–71)		
Flexible	2	97(90–100)	93(66–100)	6.6(1.9–23.7)	0.05(0.02–0.16)	235(24–2295)		
Ratio of head and uncinate								
>0.6	6	90(86–94)	96(87–100)	8.7(3.6–20.6)	0.11(0.05–0.23)	125(38–411)	0.62(0.08–4.61)	0.599
<0.6	6	86(82–90)	98(89–100)	9.2(3.4–24.9)	0.16(0.06–0.40)	62(14–272)		
No. of patinets								
>50	10	85(82-88)	98(93–100)	11.9(5.6–25.8)	0.14(0.08–0.24)	98(40–242)	0.19(0.03–1.14)	0.067
<50	6	82(75–88)	94(73–100)	4.7(1.9–11.4)	0.22(0.09–0.55)	25(7–96)		

PLR, positive likelihood ratio; NLR, Negative likelihood ratio; DOR, diagnostic odds ratio; RDOR, relative diagnostic odds ratio; CI, confidence intervals.
